# Association between transitional care in acute care hospitals and ambulatory care sensitive condition–related readmission

**DOI:** 10.1093/ageing/afaf247

**Published:** 2025-09-09

**Authors:** Ako Machida, Noriko Morioka, Mutsuko Moriwaki, Kazuhiro Abe, Chihiro Takahashi, Kenshi Hayashida, Masayo Kashiwagi

**Affiliations:** Department of Nursing Health Services Research, Graduate School of Health Care Sciences, Institute of Science Tokyo, Yushima, Bunkyo-ku, Tokyo, Japan; Department of Epidemiology and Biostatistics, National Institute of Public Health, Minami 2-3-6, Wako, Saitama, Japan; Department of Health Policy and Informatics, Graduate School of Medical and Dental Sciences, Institute of Science Tokyo, Bunkyo-ku, Tokyo, Japan; Quality Management Center, Institute of Science Tokyo, Yushima, Bunkyo-ku, Tokyo, Japan; Department of Health Care Policy, Faculty of Medicine, Hokkaido University, Sapporo, Hokkaido, Japan; Quality Management Center, Institute of Science Tokyo, Yushima, Bunkyo-ku, Tokyo, Japan; Department of Nursing Data Science, Graduate School of Medicine, The University of Tokyo, Hongo, Bunkyo-ku, Tokyo, Japan; Department of Nursing Health Services Research, Graduate School of Health Care Sciences, Institute of Science Tokyo, Yushima, Bunkyo-ku, Tokyo, Japan

**Keywords:** health services research, older adults, readmission, transitional care

## Abstract

**Background:**

Little is known about how ambulatory care sensitive condition (ACSC)–related readmissions can be reduced in acute care settings.

**Objective:**

This study examined the association between transitional care for hospitalised older patients with ACSC and ACSC-related readmissions.

**Methods:**

This retrospective observational cohort study included patients aged 65 years and older admitted with ACSC as the primary diagnosis from 1 April 2022 to 31 January 2023, using linked data from the Diagnosis Procedure Combination and the medical functions of the hospital beds database. The primary outcomes were cumulative readmissions within 1–7, 1–14, 1–21, 1–30 and 1–60 days, analysed using inverse probability treatment weighting regression models.

**Results:**

Among 85 582 patients from 711 hospitals, 39 916 (46.6%) were female, with a median age of 82 years (interquartile range: 75–88); 57 127 (66.8%) patients received transitional care. The overall readmission rates were 2.9%, 6.0%, 8.7%, 11.4% and 17.5% among total hospitalisations within 7, 14, 21, 30 and 60 days, respectively. Overall, transitional care was associated with reduced odds of ACSC-related readmission, with odds ratios ranging from 0.72 (95% CI: 0.65–0.78) within 7 days to 0.91 (95% CI: 0.87–0.95) within 60 days. The association between transitional care and readmission varied by ACSC category. In chronic ACSC, the association was strongest for 7-day readmission, followed by a downward trend. In acute and vaccine-preventable ACSC, the association was strongest for 7-day readmission but levelled off after 21 days.

**Conclusions:**

Transitional care in acute care hospitals may be associated with a reduced risk of early readmissions due to ACSC when older patients are hospitalised.

## Key Points

- Transitional care for older patients hospitalised due to ACSC was significantly associated with reduced ACSC-related readmission.
- This study shows transitional care’s potential to break the cycle of repeated hospitalisation for ACSC in older patients.
- Findings highlight collaboration’s role in improving care continuity and reducing avoidable hospitalisations in the healthcare system.

## Introduction

Hospitalisation due to an ambulatory care sensitive condition (ACSC) in the older population represents a critical global issue. ACSC serves as a key indicator of primary care quality and encompasses conditions for which timely and appropriate primary care interventions can potentially prevent hospitalisations [1, 2]. Studies have reported that ACSC hospitalisations accounted for 18.5% of all hospital admissions in England [3], 10.5% in the USA [4], 4.2% in Korea [5] and 8.4% in Japan [6].

Initiatives aimed at reducing ACSC hospitalisations have been extensively studied in primary care settings: continuous care from primary care physicians [7, 8], a higher density of primary care physicians [9] and multidisciplinary collaboration within communities [10] have been reported as effective strategies to prevent ACSC hospitalisations. Regardless of these efforts, hospitalisations due to ACSC in the older population remain a persistent global challenge [11]. In particular, older patients with ACSC repeatedly experience ACSC-related readmissions [12–14], highlighting the deep-rooted nature of the problem. To reduce recurrent hospitalisations due to ACSC, it is indispensable to strengthen transitional care from acute care hospitals to primary care while reducing readmissions among this vulnerable population.

Transitional care has been focused on and has provided accumulating evidence to reduce hospital readmissions [15–19]. Naylor *et al*. conducted a randomised controlled trial demonstrating that an advanced-practice registered nurse–led transitional care intervention for hospitalised older adults with heart failure significantly prolonged the time to readmission. It reduced the number of readmissions and healthcare costs in 1 year [20, 21]. Another randomised controlled trial by Coleman *et al*. revealed that a care transition intervention, which included a transition coach and tools to enhance patient engagement, significantly reduced rehospitalisation rates at 30, 90 and 180 days among older adults with chronic illnesses [22]. However, the association of transitional care introduced in the healthcare system has not been sufficiently evaluated. Furthermore, it is necessary to comprehensively consider diseases in which hospitalisation is, to some extent, potentially avoidable with appropriate outpatient care, such as ACSC.

Therefore, this study aimed to examine the association between transitional care in acute care hospitals and a decrease in the likelihood of readmissions due to ACSC in older patients, specifically those aged 65 years and older who were admitted with ACSC as their primary diagnosis, using the Japanese claims data.

## Methods

This retrospective cohort study was approved by the institutional review board of the Institute of Science Tokyo (approval no. M2023-113- 02). The study was conducted in accordance with the Declaration of Helsinki, and the need for informed consent was waived due to the anonymous nature of the data. Reporting followed the guidelines for the Reporting of Observational Studies in Epidemiology.

### Data source

This study used linked data from the Diagnosis Procedure Combination (DPC) database and reports on the medical functions of hospital beds nationwide in Japan. The linkage was performed using hospital IDs assigned to individual DPC records, enabling the linking of hospital characteristics obtained from the reports on the medical functions of hospital beds. The DPC payment system, introduced in 2003 for acute inpatient care, includes a fixed per diem payment based on diagnosis-related groups [23]. Acute care hospitals that adopted the DPC system have steadily increased, reaching 1786 hospitals and ~480 000 beds in fiscal year 2024, representing around 85% of the beds covered by acute care basic inpatient fees nationwide [24]. Reports on the medical functions of hospital beds [25], compiled as government statistical data and publicly available, were implemented based on the 2014 amendment to the Medical Care Act. All hospitals and clinics with general or long-term care beds are mandated to report annually to the prefectural government, such as medical functions, medical equipment status, medical treatments, number of healthcare professionals, patient admissions and discharge status.

### Study population

Participants aged 65 years and older were admitted with ACSC as the primary diagnosis and discharged between 1 April 2022 and 31 January 2023. Exclusion criteria were planned hospitalisation, hospitalisation in the 6 months before admission, a length of stay of ≤2 days and in-hospital death. To improve comparability between groups, patients with frequent hospitalisations were excluded, as they likely had substantially different characteristics, limiting the validity of propensity score adjustment. A length of stay of ≤2 days was deemed too short to enable the implementation of transitional care. In particular, under the reimbursement system, screening inpatients with difficulties in the transition to the post-discharge setting (called ‘high-risk patients’) is typically identified within 3–7 days of admission. Since the definition of ACSC is not yet established in Japan, this study used the definitions described by Purdy *et al*. in their research on ACSC [2]. ACSC was classified into three categories: acute ACSC, which can prevent acute exacerbations with effective management; chronic ACSC, where early intervention can prevent the progression to more severe conditions; and vaccine-preventable ACSC, which can minimise the incidence of specific diseases through vaccination. The study included 158 diseases or conditions based on the International Statistical Classification of Diseases-10 codes (ICD-10) from the DPC data of patients [26].

### Outcome

The outcome variables were cumulative readmissions within 1–7, 1–14, 1–21, 1–30 and 1–60 days, assuming that readmissions for a series of conditions occur in the same hospital. If the patient had multiple readmissions, only the first readmission was included. Since a single patient may have multiple ACSCs, the ACSC for the initial hospitalisation and for readmission did not need to be the same. For instance, a patient might be initially hospitalised for dehydration and later readmitted for congestive heart failure.

### Transitional care

As the independent variable, receiving transitional care during hospitalisation was defined as present if any of the following services were provided: discharge planning service; a pre-discharge conference among hospital professionals, home-based healthcare providers (e.g. primary care physicians, visiting doctors, home health nurses) and/or care managers in the long-term care insurance; medical information sharing between doctors in the hospital and primary care physicians or visiting doctors; and home health care instruction fees.

In Japan, transitional care services are in the fee schedule of the National Health Insurance. The central component of the transitional care services in the fee schedule is the discharge planning fee, which was newly established in the 2008 revision of medical reimbursement [27]. The discharge planning is delivered by nurses in the ward where the patient is hospitalised and by the nurse or medical social worker (MSW) in the discharge planning department in the hospital. It includes screening high-risk patients, holding multidisciplinary meetings for the high-risk patients, supporting the patients and their caregivers’ decision-making and sharing information with professionals in the next care settings. The details of other transitional care services are presented in Table 1.

**Table 1:** Details of transitional care services in the fee schedule of the National Health Insurance

Item name	Year of introduction	Description	Person in charge
Home healthcare instruction fees	1991	The physician must provide documentation regarding home healthcare for patients who cannot visit outpatient facilities due to illness or injury. The text includes basic information about the patient, their current condition, activities of daily living, red flag signs and symptoms and contact information. This fee can be claimed once a month.	Physician
Discharge planning fee	2008	A DPD must be established with at least one dedicated nurse and one MSW. Discharge Planning Fee Type 1 requires one dedicated staff member for discharge planning support for every two wards, and high-risk patients must be identified within 3 days of admission. Discharge Planning Fee Type 2 does not have staffing requirements but requires high-risk patients to be identified within 7 days of admission. This fee can be claimed once at the time of discharge.	Nurses and MSWs in the DPD
Pre-discharge conference among hospital professionals, home-based healthcare providers and/or care managers in the LTCI	2010	The hospital professionals, home-based healthcare providers and/or care managers in the LTCI collaborate to provide the patient and their family with detailed explanations of the treatment progress, assess their understanding and confirm the necessary services and self-care instructions required post-discharge, either in person or online. Additionally, they engage in discussions to delineate the roles and responsibilities of healthcare providers between the hospital and the primary care setting. Conferences offer incentives to both hospital and community-based providers, and, if more than three healthcare professionals participate, an additional incentive will be provided. This fee can be claimed once during hospitalisation.	Nurses and MSWs in the DPD
Medical information document fee Type 3	2020	The fee can be calculated when hospitals provide written feedback about the medical treatment of a patient referred by a primary care doctor. This fee can be claimed once every 3 months.	Referred physician

DPD, discharge planning department; MSW, medical social worker; LTCI, long-term care insurance.

In order to claim these items, care plans and documentation of interventions are required to be recorded in the patient’s medical records.

### Adjusted variables

Adjusted variables were selected considering previous research [28, 29] and clinical significance. Patient characteristics were as follows: sex, age, body mass index (BMI), Charlson Comorbidity Index (CCI; 0, 1, 2 and 3 points or more based on ICD-10 Coding Algorithms for Charlson Comorbidities) [30], the presence of dementia, the presence of dialysis, admission to the emergency room (ER) or intensive care unit (ICU), use of a ventilator, prescription of injectable antidiabetic medications (insulin or glucagon-like peptide-1 receptor agonists), use of any type of home care before hospitalisation, the application for long-term care insurance and location before hospitalisation. We extracted medications corresponding to pancreatic hormones from the Anatomical Therapeutic Chemical (ATC) Classification System codes among injectable antidiabetic medications (Table S1). For dementia status, patients who were classified as Level I or higher on the ‘Daily Living Independence Assessment for Elderly Individuals with Dementia’ tool [31], indicating any dementia, and who had been prescribed dementia medication based on ATC Classification System codes (Table S1), were considered to have dementia. Hospital characteristics were all obtained from the 2022 reports on the medical functions of hospital beds [25]: the type of ownership, regional medical care support hospital approval, home medical care support hospital approval, the number of hospital beds, the number of full-time equivalent (FTE) hospital physicians per 100 hospital beds, the number of FTE nursing staff per 100 hospital beds and the number of FTE nurses and MSWs in the discharge planning department per 100 hospital beds. The regional characteristics were selected at the secondary medical area units from publicly administrative data: population and the proportion of people aged 65 years and older [32], the number of home care support clinics per 10 000 people aged 65 years and older [33] and the number of in-home service agencies per 10 000 people aged 65 years and older [34]. The secondary medical area is defined as a unit where healthcare can be provided comprehensively within the region, and it is used as a unit of investigation for regional healthcare resources [35].

### Statistical analysis

Cases other than ACSC, hospitals that did not meet the legal standards and cases with missing values for covariates were excluded, and only complete cases were analysed. First, descriptive statistics were calculated using the median and interquartile range (IQR) for continuous variables and frequency and proportions for categorical variables. Percentages of overall readmission due to ACSC within 7, 14, 21, 30 and 60 days by transitional care are depicted in bar graphs. Second, the propensity score (PS) method was used to control selection bias due to baseline characteristics [36–38]. The PS, the probability of receiving transitional care, was generated for each patient by a logistic regression model with transitional care as the dependent variable and the aforementioned covariates as independent variables. The c-statistics were estimated, and the overlap of the PS distribution was checked. The association between transitional care and readmission within 7, 14, 21, 30 and 60 days was estimated in a logistic analysis using the inverse probability treatment weighting (IPTW) approach, incorporating stabilised average treatment effect weights to prevent unstable effect estimation due to excessively large or small weighting values. The balance of covariates was assessed before and after PS weighting by calculating standard differences (SDs), with an SD < 0.1 (10%) considered acceptable. To assess the potential effect of unmeasured confounding, the E-value was calculated [39].

The significance level was set at a two-tailed *P* < 0.05. All analyses were performed using Stata version 18.0 (Stata Corp., College Station, TX, USA).

## Results

**Figure 1:**
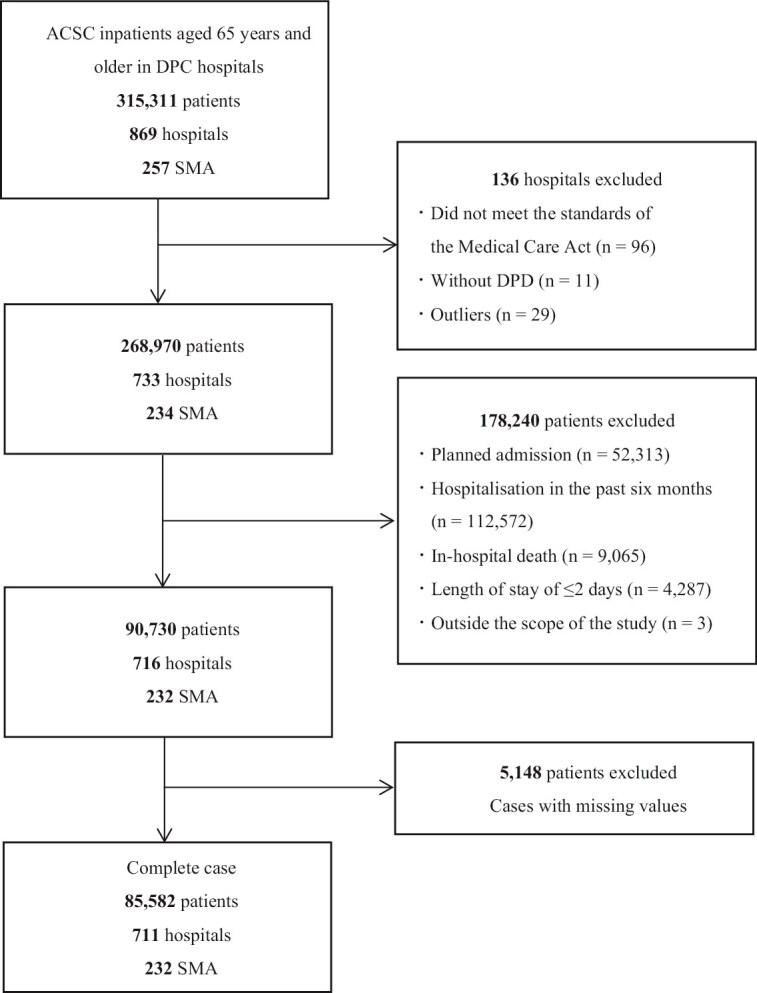
Flowchart of sample selection. ACSC, ambulatory care sensitive condition; SMA, secondary medical area; DPD, discharge planning department.

In total, 85 582 patients from 711 hospitals across 232 secondary medical areas were included in the analysis (Figure 1). By the ACSC category, chronic ACSC accounted for the highest proportion, with 48 116 patients (56.2%), followed by acute ACSC with 28 315 patients (33.1%) and vaccine-preventable ACSC with 9151 patients (10.7%) (Table 2). According to the ICD-10 codes, congestive heart failure was the most common diagnosis at 37.1%, followed by pyelonephritis at 13.3% and influenza or pneumonia at 10.7% (Table S2).

**Table 2:** Baseline characteristics before and after inverse probability treatment weighting (*N* = 85 582)

		Before weighted			After weighted		
	Overall	With transitional care	Without transitional care	Standard difference	With transitional care	Without transitional care	Standard difference
		(*n* = 57 127)	(*n* = 28 455)		(*n* = 57 127)	(*n* = 28 455)	
ACSC category, *n* (%)							
Acute	28 315 (33.1)	18 540 (32.5)	9775 (34.4)	0.04	18,773 (32.9)	9949 (33.4)	0.00
Chronic	48 116 (56.2)	32 598 (57.1)	15 518 (54.5)	0.05	32 298 (56.5)	15 831 (55.6)	0.00
Vaccine-preventable	9151 (10.7)	5989 (10.5)	3162 (11.1)	0.02	6055 (10.6)	3125 (11.0)	0.00
Female, *n* (%)	39 916 (46.6)	27 822 (48.7)	12 094 (45.5)	0.12	26 657 (46.7)	13 334 (46.9)	0.00
Age, median (IQR)	82 (75–88)	83 (77–89)	80 (74–86)	0.35	82 (75–88)	82 (75–88)	0.02
Location before hospitalisation, *n* (%)							
Home	74 694 (87.3)	48 809 (85.4)	25 885 (91.0)	0.17	49 786 (87.2)	24 624 (86.6)	0.02
Transfer	1865 (2.2)	1325 (2.3)	531 (1.9)	0.03	1239 (2.2)	641 (2.3)	0.01
Long-term care facility	8981 (10.5)	6956 (12.2)	2025 (7.1)	0.17	6068 (10.6)	3172 (11.2)	0.01
Other	51 (0.1)	37 (0.1)	14 (0.1)	0.01	34 (0.1)	18 (0.1)	0.01
Body mass index, median (IQR)	22 (19.0–24.3)	21 (18.7–24.1)	22 (19.5–24.7)	0.14	22 (18.9–24.3)	22 (19.1–24.3)	0.01
Charlson Comorbidity Index, *n* (%)							
0 points	18 847 (22.0)	11 361 (19.9)	7486 (26.3)	0.15	12 147 (21.3)	6498 (22.8)	0.01
1 point	7475 (8.7)	4603 (8.1)	2872 (10.1)	0.07	4970 (8.7)	2514 (8.8)	0.00
2 points	31 450 (36.8)	21 478 (37.6)	9972 (35.0)	0.05	21 392 (37.5)	10 116 (35.6)	0.00
3 points or more	27 810 (32.5)	19 685 (34.5)	8125 (28.6)	0.13	18 617 (32.6)	9328 (32.8)	0.00
Presence of dementia, *n* (%)	13 681 (16.0)	10 852 (19.0)	2829 (9.9)	0.26	9208 (16.1)	4850 (17.1)	0.03
Use of ER or ICU, *n* (%)	14 279 (16.7)	10 268 (18.0)	4011 (14.1)	0.12	9534 (16.7)	4749 (17.0)	0.00
Use of ventilator, *n* (%)	765 (0.9)	556 (1.0)	209 (0.7)	0.03	514 (0.9)	265 (0.9)	0.00
Use of dialysis, *n* (%)	2724 (3.2)	1643 (2.9)	1081 (3.8)	0.05	2729 (9.6)	1819 (3.2)	0.03
Prescription of injectable antidiabetic medication, *n* (%)	7985 (9.3)	5590 (9.8)	2395 (8.4)	0.05	5341 (9.4)	2729 (9.6)	0.01
Home care before hospitalisation, *n* (%)							
No	75 872 (88.7)	49 243 (86.2)	26 629 (93.6)	0.25	50 602 (88.6)	25 053 (88.1)	0.02
Yes	8710 (10.2)	7009 (12.3)	1701 (6.0)	0.23	5859 (10.3)	3082 (10.8)	0.02
Unknown	1000 (1.2)	875 (1.5)	125 (0.4)	0.11	666 (1.2)	320 (1.1)	0.00
Status of long-term care insurance, *n* (%)							
None	43 018 (50.3)	24 411 (42.7)	18 607 (42.7)	0.47	28 633 (50.1)	14 025 (49.3)	0.02
In application or certified	40 672 (47.5)	31 539 (55.2)	9133 (32.1)	0.48	27 240 (47.7)	13 807 (48.5)	0.02
Unknown	1892 (2.2)	1177 (2.1)	715 (2.5)	0.03	1254 (2.2)	623 (2.2)	0.00
Type of ownership, *n* (%)							
National	11 043 (12.9)	7125 (12.5)	3918 (13.8)	0.04	7409 (13.0)	3719 (13.1)	0.00
Public	38 609 (45.1)	27 907 (48.9)	10 702 (37.6)	0.23	25 737 (45.1)	12 747 (44.8)	0.01
Social	2286 (2.7)	1638 (2.9)	648 (2.3)	0.04	1511 (2.7)	746 (2.6)	0.00
Private	29 904 (34.9)	18 483 (32.4)	11 421 (40.1)	0.16	19 919 (34.9)	9971 (35.0)	0.00
Others	3740 (4.4)	1974 (3.5)	1766 (6.2)	0.12	2551 (4.5)	1272 (4.5)	0.00
Accreditation as a regional medical care support hospital, *n* (%)	54 726 (64.0)	39 010 (68.3)	15 716 (55.2)	0.27	36 517 (63.9)	18 158 (63.8)	0.00
Accreditation as a home medical care support hospital, *n* (%)	21 600 (25.2)	15 183 (25.6)	6417 (22.6)	0.09	14 451 (25.3)	7280 (25.6)	0.01
Number of hospital beds, median (IQR)	419 (301–570)	424 (311–570)	405 (292–570)	0.02	418 (304–570)	414 (300–570)	0.01
Number of doctors per 100 beds, median (IQR)	28 (20.2–36.1)	28 (20.4–35.7)	27 (19.7–36.7)	0.06	28 (20.2–36.1)	27 (19.9–35.4)	0.01
Number of nursing staff per 100 beds, median (IQR)	102.7 (89.8–114.4)	102 (90.0–114.2)	103 (89.2–115.7)	0.02	102 (89.8–114.2)	103 (89.3–114.5)	0.00
Number of nursing staff and MSWs in discharge planning department per 100 beds, median (IQR)	2.5 (1.9–3.4)	2.6 (2.0–3.4)	2.4 (1.8–3.4)	0.08	2.5 (1.9–3.4)	2.5 (1.8–3.5)	0.01
Population (100 000 people), median (IQR)	5.9 (2–14.8)	6.3 (2.0–14.7)	5.7 (2.0–14.7)	0.01	6.7 (2.1–14.7)	5.9 (2.0–14.7)	0.01
Percentage of individuals aged 65 years and older, median (IQR)	30.7 (26.8–36.2)	30.1 (26.6–36.2)	31.1 (26.8–36.2)	0.10	30.6 (26.4–36.2)	31.0 (26.8–36.2)	0.01
Number of home healthcare support clinics per 10 000 people aged 65 years and older, median (IQR)	3.1 (1.1–11.7)	3.3 (1.1–12.0)	3.0 (1.1–8.6)	0.10	3.1 (1.1–11.7)	3.1 (1.1–11.4)	0.01
Number of in-home service agencies per 10 000 people aged 65 years and older, median (IQR)	216.8 (93.4–654.5)	216.8 (93.3–654.5)	215.4 (93.3–654.5)	0.04	215.5 (94.3–654.5)	216.8 (93.3–654.5)	0.01

IQR, interquartile range; ER, emergency room; ICU, intensive care unit; MSW, medical social worker.

Overall, 39 916 (46.6%) were females, and the median age was 82 years (IQR, 75–88), with 57 127 (66.8%) patients receiving transitional care. Before weighting, patients receiving transitional care were more likely to be admitted from long-term care facilities; use home care services; have applied for long-term care insurance; and have lower BMI, higher CCI scores, dementia and severe conditions requiring ER or ICU admission, as well as use respiratory treatments or injectable antidiabetic medications. After weighting, all baseline characteristics had standardised differences of <0.1, indicating that they were well balanced. The c-statistic for the PS model was 0.70. The details of the characteristics by ACSC category are shown in Tables S3–S5.

**Figure 2:**
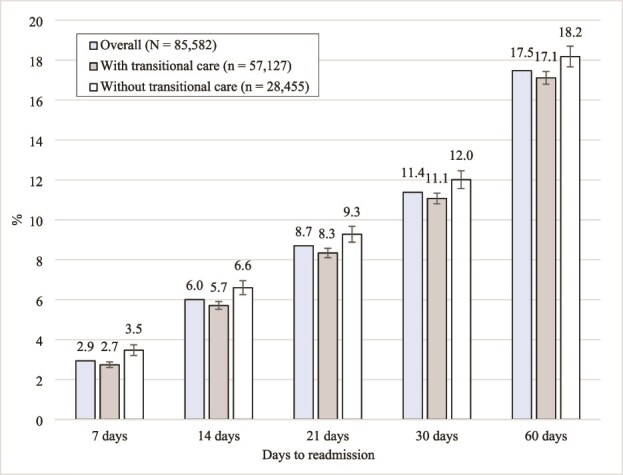
Actual overall readmission rates by number of days after discharge (*N* = 85 582). The error bars represent the 95% confidence intervals for the adjusted predicted percentages, estimated using an inverse probability treatment weighting model.

For all cutoffs, the readmission rates were lower with transitional care than without transitional care (Figure 2). The actual readmission rates with transitional care were 2.7%, 5.7%, 8.3%, 11.1% and 17.1% within 7, 14, 21, 30 and 60 days, respectively. In contrast, the predicted readmission rates, estimated by using the IPTW model, slightly decreased by 0.04 points (Table S6). The actual readmission rates without transitional care were 3.5%, 6.6%, 9.3%, 12.0% and 18.2% within 7, 14, 21, 30 and 60 days, respectively, while the predicted rates increased by 0.3–0.4 points. These results showed similar trends across all ACSC categories (Table S6). The readmission rates for the transitional care group were estimated to be lower as confounding factors were controlled, whereas the rates for the group without transitional care were higher after adjusting for confounding factors.

**Figure 3:**
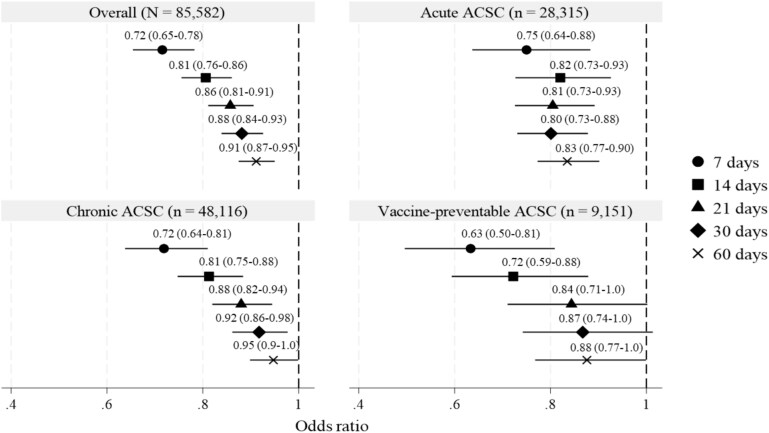
Results of inverse probability treatment weighting analysis for the association between transitional care and readmission. Analysis was conducted using the following adjusting variables for all the categories: sex, age, location before hospitalisation, body mass index, Charlson Comorbidity Index, presence of dementia, use of emergency room or intensive care unit, use of a ventilator, use of dialysis, prescription of injectable antidiabetic medication, home care before hospitalisation, the status of long-term care insurance, type of ownership, accreditation as a regional medical care support hospital, accreditation as a home medical care support hospital, number of hospital beds, number of doctors per 100 hospital beds, number of nursing staff per 100 hospital beds, number of nursing staff and medical social workers in discharge planning department per 100 hospital beds, population (100 000 people), percentage of individuals aged 65 years and older, number of home health care support clinics per 10 000 people aged 65 years and older and number of in-home service agencies per 10 000 people aged 65 years and older. The parentheses indicate the 95% confidence interval.

The IPTW regression analysis examining the association between transitional care and readmission related to ACSC is as follows: The odds ratios (ORs) for overall readmission within 7, 14, 21, 30 and 60 days were 0.72 (95% confidence interval (CI): 0.65–0.78, *P* < 0.001), 0.81 (95% CI: 0.76–0.86, *P* < 0.001), 0.86 (95% CI: 0.81–0.91, *P* < 0.001), 0.88 (95% CI: 0.84–0.93, *P* < 0.001), 0.91 (95% CI: 0.87–0.95, *P* < 0.001), respectively (Figure 3). The association of transitional care varied by ACSC category and cutoffs. For acute ACSC, the ORs ranged from 0.75 within 7 days (95% CI: 0.64–0.88, *P* < 0.001) to 0.83 within 60 days (95% CI: 0.77–0.90, *P* < 0.001), maintaining an OR consistently in the 0.8 range from 14 to 60 days post-discharge. In chronic ACSC, the ORs ranged from 0.72 within 7 days (95% CI: 0.64–0.81, *P* < 0.001) to 0.95 within 60 days (95% CI: 0.90–1.0, *P* < 0.001). While significant reductions were observed in the shorter periods, the decrease became less pronounced over time. For vaccine-preventable ACSC, the ORs ranged from 0.63 within 7 days (95% CI: 0.50–0.81, *P* < 0.001) to 0.88 within 60 days (95% CI: 0.77–1.0, *P* < 0.001). The reduction in readmission risk was less consistent, with a downward trend observed at 21 days (OR: 0.84, 95% CI: 0.71–1.0, *P* = 0.05) and 30 days (OR: 0.87, 95% CI: 0.74–1.0, *P* = 0.07).

## Discussion

To our knowledge, this is the first study to find that transitional care in acute care hospitals can significantly reduce ACSC-related readmissions among older adults with ACSC. This finding, however, is consistent with previous studies on different populations, such as those with ACSC-defined conditions [40], older medical patients [41] and older adults with frailty [42].

In previous studies that targeted patients hospitalised for pneumonia, congestive heart failure or chronic obstructive pulmonary disease exacerbation, transitional care provided by nurse transitional care coordinators resulted in significantly lower odds of readmission at 30 days (OR = 0.512, 95% CI 0.392–0.668) [40]. Another systematic review for older medical patients reported that 22 of 29 transitional care interventions resulted in a drop in readmissions [41]. Moreover, an umbrella review for older adults with frailty and readmission showed a reduction in high-intensity transitional care interventions [42]. As a possible mechanism, the transitional care provided in acute care hospitals contributes to sustaining the continuity of connection among hospitals, next-care settings and the patient from admission to discharge and beyond. In this regard, transitional care coordinated with general practitioners or community nurses [43, 44] and continuity of care, which includes relational, informational and management aspects [45], have been reported to contribute to reduced rehospitalisation. This study not only strengthens the evidence by comprehensively examining ACSC, a group of conditions potentially avoidable through outpatient care, using real-world data, but also highlights the essential linkage needed to promote close communication extending beyond the acute phase of illness over time.

The pattern of association was found to vary by the ACSC category when the cut-off points were adjusted to 7, 14, 21, 30 and 60 days. This variation might be due to the nature of the underlying condition. In the chronic ACSC, the association of transitional care showed a downward trend, with the highest association observed for 7-day readmission. The most common condition in this study, congestive heart failure, follows a trajectory of recurrent hospitalisations [46, 47]. Expanding the cutoff range likely increases the odds while strengthening the connection among the hospital, primary care settings and the patient to facilitate timely hospitalisation. In acute and vaccine-preventable ACSC, the declines were most pronounced for 7-day readmission, but the trend levelled off after 21 days. Since acute and vaccine-preventable ACSC are predominantly sudden-onset inflammatory conditions without underlying chronic diseases, readmission is unlikely unless early discharge results in suboptimal treatment.

## Limitations

The findings should be interpreted with caution in terms of generalisability. Although transitional care is widely implemented, patients with ACSC are not always a specific target population. Thus, these results may provide valuable insights for contexts in which transitional care is less established. While the observed effect size was modest, the absolute reduction in readmission rates (0.8%–1.1%) among 85 582 older patients with ACSC in this study corresponds to a reduction of ~8000–10 000 readmissions per year in Japan, which could have meaningful implications at the national level.

Several data-related limitations should be acknowledged. Readmissions were only captured within the same hospital, which may have led to an underestimation of actual readmission rates. Which components of transitional care were most beneficial was not determined, and the 10-month data window may have missed seasonal trends. Lastly, although IPTW was used to control for confounding, residual bias remains possible. Lower E-values at longer time points suggest that unmeasured factors, such as socioeconomic status, may still have influenced the outcomes (Table S7) [48–50]. Furthermore, we could not account for the hierarchical structure of the data (patient, hospital and regional levels), which may have introduced additional unexplained variation.

To enhance our understanding of transitional care, further research should examine case-specific components, intervention duration tailored to ACSC categories and interprofessional collaboration across hospital and community settings, including the post-discharge period.

## Conclusion

Among older ACSC adults admitted to acute care hospitals, transitional care during hospitalisation may have helped reduce the risk of readmissions due to ACSC. The association varied across different ACSC categories. To mitigate the risk of a vicious cycle of recurrent hospitalisations due to ACSC, ensuring seamless communication among acute care hospitals, the next care setting and the patient is essential for the continuity of care.

## Supplementary Material

aa-25-1112-File002_afaf247

## Research Data Transparency and Availability

Due to the nature of the data used in this study (anonymised administrative data), the datasets are not publicly available. The data are available from the corresponding author upon reasonable request and with permission from the data provider.

## References

[1] AHRQ quality indicators—guide to prevention quality indicators: hospital admission for ambulatory care sensitive conditions. Rockville, MD: Agency for Healthcare Research and Quality, 2001. AHRQ. Pub. No. 02-R0203.

[2] Purdy S, Griffin T, Salisbury C et al. Ambulatory care sensitive conditions: terminology and disease coding need to Be more specific to aid policy makers and clinicians. Public Health 2009;123:169–73. 10.1016/j.puhe.2008.11.001. 19144363

[3] Bardsley M, Blunt I, Davies S et al. Is secondary preventive care improving? Observational study of 10-year trends in emergency admissions for conditions amenable to ambulatory care. BMJ Open 2013;3:e002007. 10.1136/bmjopen-2012-002007. PMC3549201 23288268

[4] Loyd C, Blue K, Turner L et al. National Norms for hospitalisations due to ambulatory care sensitive conditions among adults in the US. J Gen Intern Med 2023;38:2953–9. 10.1007/s11606-023-08161-z. 36941421 PMC10027258

[5] Park H, Son MJ, Jung DW et al. National trends in hospitalisation for ambulatory care sensitive conditions among Korean adults between 2008 and 2019. Yonsei Med J 2022;63:948–55. 10.3349/ymj.2022.0110. 36168248 PMC9520050

[6] Iba A, Tomio J, Abe K et al. Hospitalisations for ambulatory care sensitive conditions in a large City of Japan: a descriptive analysis using claims data. J Gen Intern Med 2022;37:3917–24. 10.1007/s11606-022-07713-z. 35829872 PMC9640483

[7] Nyweide DJ, Anthony DL, Bynum JPW et al. Continuity of care and the risk of preventable hospitalisation in older adults. JAMA Intern Med 2013;173:1879–85. 10.1001/jamainternmed.2013.10059. 24043127 PMC3877937

[8] Barker I, Steventon A, Deeny SR. Association between continuity of care in general practice and hospital admissions for ambulatory care sensitive conditions: cross sectional study of routinely collected, person level data. BMJ 2017;356:j84. 10.1136/bmj.j84. 28148478

[9] Lin YH, Eberth JM, Probst JC. Ambulatory care–sensitive condition hospitalisations among Medicare beneficiaries. Am J Prev Med 2016;51:493–501. 10.1016/j.amepre.2016.05.005. 27374209

[10] Duminy L, Ress V, Wild EM. Complex community health and social care interventions – which features lead to reductions in hospitalisations for ambulatory care sensitive conditions? A systematic literature review. Health Policy 2022;126:1206–25. 10.1016/j.healthpol.2022.10.003. 36257866

[11] Weeks WB, Ventelou B, Paraponaris A. Rates of admission for ambulatory care sensitive conditions in France in 2009-2010: trends, geographic variation, costs, and an international comparison. Eur J Health Econ 2016;17:453–70. 10.1007/s10198-015-0692-y. 25951924

[12] Sarmento J, Santana R. Multiple admissions for ambulatory care sensitive conditions: target for intervention? Int J Integr Care 2016;16:A235. 10.5334/ijic.2783.

[13] Agana DFG, Striley CW, Cook RL et al. A novel approach to characterizing readmission patterns following hospitalisation for ambulatory care-sensitive conditions. J Gen Intern Med 2020;35:1060–8. 10.1007/s11606-020-05643-2. 31993948 PMC7174498

[14] Hirota Y, Kunisawa S, Fushimi K et al. Association between clinic physician workforce and avoidable readmission: a retrospective database research. BMC Health Serv Res 2020;20:125. 10.1186/s12913-020-4966-4. 32070343 PMC7029440

[15] Burke RE, Guo R, Prochazka AV et al. Identifying keys to success in reducing readmissions using the ideal transitions in care framework. BMC Health Serv Res 2014;14:423. 10.1186/1472-6963-14-423. 25244946 PMC4180324

[16] Kripalani S, Theobald CN, Anctil B et al. Reducing hospital readmission rates: current strategies and future directions. Annu Rev Med 2014;65:471–85. 10.1146/annurev-med-022613-090415. 24160939 PMC4104507

[17] Leppin AL, Gionfriddo MR, Kessler M et al. Preventing 30-day hospital readmissions: a systematic review and meta-analysis of randomized trials. JAMA Intern Med 2014;174:1095–107. 10.1001/jamainternmed.2014.1608. 24820131 PMC4249925

[18] Le Berre M, Maimon G, Sourial N et al. Impact of transitional Care Services for Chronically ill older patients: a systematic evidence review. J Am Geriatr Soc 2017;65:1597–608. 10.1111/jgs.14828. 28403508

[19] Soh YY, Zhang H, Toh JJY et al. The effectiveness of tele-transitions of care interventions in high-risk older adults: a systematic review and meta-analysis. Int J Nurs Stud 2023;139:104428. 10.1016/j.ijnurstu.2022.104428. 36682322

[20] Naylor MD, Brooten D, Campbell R et al. Comprehensive discharge planning and home follow-up of hospitalized elders: a randomized clinical trial. JAMA 1999;281:613–20. 10.1001/jama.281.7.613. 10029122

[21] Naylor MD, Brooten DA, Campbell RL et al. Transitional care of older adults hospitalized with heart failure: a randomized, controlled trial. J Am Geriatr Soc 2004;52:675–84. 10.1111/j.1532-5415.2004.52202.x. 15086645

[22] Coleman EA, Parry C, Chalmers S et al. The care transitions intervention: results of a randomized controlled trial. Arch Intern Med 2006;166:1822–8. 10.1001/archinte.166.17.1822. 17000937

[23] Hayashida K, Murakami G, Matsuda S et al. History and profile of diagnosis procedure combination (DPC): development of a real data collection system for acute inpatient Care in Japan. J Epidemiol 2021;31:1–11. 10.2188/jea.JE20200288. 33012777 PMC7738645

[24] FY2024 revision of medical fees. Hospitalisation V (DPC/PDPS, short-stay surgery, etc). Ministry of Health, Labour and Welfare. Available at: https://www.mhlw.go.jp/stf/seisakunitsuite/bunya/0000196352_00012.html. Accessed August 3, 2024.

[25] Reports on the medical function of hospital beds. Ministry of Health, Labour and Welfare. Available at: https://www.mhlw.go.jp/stf/seisakunitsuite/bunya/0000055891.html. Accessed August 3, 2024.

[26] Abe K, Kawachi I, Iba A et al. In-hospital deaths from ambulatory care–sensitive conditions before and during the COVID-19 pandemic in Japan. JAMA Netw Open 2023;6:e2319583. 10.1001/jamanetworkopen.2023.19583. 37347480 PMC10288336

[27] Discharge Coordination (Medical Care Coordination) . Ministry of health, labour and welfare. 2011; Available at: https://www.mhlw.go.jp/stf/shingi/2r98520000011ga6-att/2r98520000011gkm.pdf. Accessed August 3, 2024.

[28] Van Walraven C, Dhalla IA, Bell C et al. Derivation and validation of an index to predict early death or unplanned readmission after discharge from hospital to the community. CMAJ 2010;182:551–7. 10.1503/cmaj.091117. 20194559 PMC2845681

[29] Kansagara D, Englander H, Salanitro A et al. Risk prediction models for hospital readmission: a systematic review. JAMA 2011;306:1688–98. 10.1001/jama.2011.1515. 22009101 PMC3603349

[30] Quan H, Sundararajan V, Halfon P et al. Coding algorithms for defining comorbidities in ICD-9-CM and ICD-10 administrative data. Med Care 2005;43:1130–9. 10.1097/01.mlr.0000182534.19832.83. 16224307

[31] Kuroda N, Hamada S, Sakata N et al. Antipsychotic use and related factors among people with dementia aged 75 years or older in Japan: a comprehensive population-based estimation using medical and long-term care data. Int J Geriatr Psychiatry 2019;34:472–9. 10.1002/gps.5041. 30478985 PMC6590349

[32] Resident Basic Registry . E-stat statistics of Japan. 2022; Available at: https://www.e-stat.go.jp/. Accessed August 3, 2024.

[33] Regional Data Collection from Home Health Care . Ministry of Health, Labour and Welfare. Available at: https://www.mhlw.go.jp/stf/seisakunitsuite/bunya/0000061944.html. Accessed August 3, 2024.

[34] Open data of the system data for the publication of nursing care service information. Ministry of Health, Labour and Welfare Available at: https://www.mhlw.go.jp/stf/kaigo-kouhyou_opendata.html. Accessed August 3, 2024.

[35] Morioka N, Tomio J, Seto T et al. The association between higher nurse staffing standards in the fee schedules and the geographic distribution of hospital nurses: a cross-sectional study using Nationwide administrative data. BMC Nurs 2017;16:25. 10.1186/s12912-017-0219-1. 28546786 PMC5442664

[36] Austin PC, Stuart EA. Moving towards best practice when using inverse probability of treatment weighting (IPTW) using the propensity score to estimate causal treatment effects in observational studies. Stat Med 2015;34:3661–79. 10.1002/sim.6607. 26238958 PMC4626409

[37] Ali MS, Prieto-Alhambra D, Lopes LC et al. Propensity score methods in health technology assessment: principles, extended applications, and recent advances. Front Pharmacol 2019;10:973. 10.3389/fphar.2019.00973. 31619986 PMC6760465

[38] Desai RJ, Franklin JM. Alternative approaches for confounding adjustment in observational studies using weighting based on the propensity score: a primer for practitioners. BMJ 2019;367:l5657. 10.1136/bmj.l5657. 31645336

[39] VanderWeele TJ, Ding P. Sensitivity analysis in observational research: introducing the E-value. Ann Intern Med 2017;167:268–74. 10.7326/M16-2607. 28693043

[40] Kripalani S, Chen G, Ciampa P et al. A transition care coordinator model reduces hospital readmissions and costs. Contemp Clin Trials 2019;81:55–61. 10.1016/j.cct.2019.04.014. 31029692 PMC6559370

[41] Fønss Rasmussen L, Grode LB, Lange J et al. Impact of transitional care interventions on hospital readmissions in older medical patients: a systematic review. BMJ Open 2021;11:e040057. 10.1136/bmjopen-2020-040057. PMC7799140 33419903

[42] Joo JY, Liu MF. Transitional care interventions for supporting frail older adults discharged from hospitals: an umbrella review. Geriatr Nurs 2023;50:80–9. 10.1016/j.gerinurse.2022.12.021. 36669435

[43] Tu Q, Xiao LD, Ullah S et al. A transitional care intervention for hypertension control for older people with diabetes: a cluster randomized controlled trial. J Adv Nurs 2020;76:2696–708. 10.1111/jan.14466. 32744373

[44] Berthelsen C, Møller N, Bunkenborg G. Transitional care model for older adults with multiple chronic conditions: an evaluation of benefits utilising an umbrella review. J Clin Nurs 2024;33:481–96. 10.1111/jocn.16913. 38108223

[45] Facchinetti G, ’Angelo D D, Piredda M et al. Continuity of care interventions for preventing hospital readmission of older people with chronic diseases: a meta-analysis. Int J Nurs Stud 2020;101:103396. 10.1016/j.ijnurstu.2019.103396. 31698168

[46] Dharmarajan K, Hsieh AF, Kulkarni VT et al. Trajectories of risk after hospitalisation for heart failure, acute myocardial infarction, or pneumonia: retrospective cohort study. BMJ 2015;350:h411. 10.1136/bmj.h411. 25656852 PMC4353309

[47] Kaneko H, Itoh H, Yotsumoto H et al. Association between the number of hospital admissions and in-hospital outcomes in patients with heart failure. Hypertens Res 2020;43:1385–91. 10.1038/s41440-020-0505-2. 32655133

[48] Billings J, Zeitel L, Lukomnik J et al. Impact of socioeconomic status on hospital use in new York City. Health Aff (Millwood) 1993;12:162–73. 10.1377/hlthaff.12.1.162. 8509018

[49] Roos LL, Walld R, Uhanova J et al. Physician visits, hospitalisations, and socioeconomic status: ambulatory care sensitive conditions in a Canadian setting. Health Serv Res 2005;40:1167–85. 10.1111/j.1475-6773.2005.00407.x. 16033498 PMC1361193

[50] Wallar LE, De Prophetis E, Rosella LC. Socioeconomic inequalities in hospitalisations for chronic ambulatory care sensitive conditions: a systematic review of peer-reviewed literature, 1990-2018. Int J Equity Health 2020;19:60. 10.1186/s12939-020-01160-0. 32366253 PMC7197160

