# Supplementary material for: Association between transitional care in acute care hospitals and ambulatory care sensitive condition–related readmission
Source: Age Ageing. 2025 Sep 9;54(9):afaf247. doi: 10.1093/ageing/afaf247 (PMC12418957; doi:10.1093/ageing/afaf247)
Supplement: aa-25-1112-File002_afaf247 [file aa-25-1112-file002_afaf247.docx]

**Association between transitional care in acute care hospitals and ambulatory care sensitive condition-related readmission**

**List of Supplemental material**

Table S1. Anatomical therapeutic chemical list

Table S2. The composition of ambulatory care sensitive condition of patients aged 65 and older (N=85,582)

Table S3. Characteristics of acute ambulatory care sensitive condition before and after inverse-probability treatment weighting (n=28,315)

Table S4. Characteristics of chronic ambulatory care sensitive condition before and after inverse-probability treatment weighting (n=48,116)

Table S5. Characteristics of vaccine-preventable ambulatory care sensitive condition before and after inverse-probability treatment weighting (n=9,151)

Table S6. Actual and predicted readmission rates by ambulatory care sensitive condition category (N=85,582)

Table S7. E-value for readmission rates due to overall ambulatory care sensitive condition (N=85,582)

Table S1. Anatomical therapeutic chemical list

| Drug classification | ATC code name | ATC code |
| --- | --- | --- |
| Antidiabetic drugs | insulin (human) | A10AB01 |

| (Injectable only) | insulin (human) | A10AC01 |
| --- | --- | --- |
|  | insulin (human) | A10AD01 |
|  | insulin aspart | A10AB05 |
|  | insulin aspart | A10AD05 |
|  | insulin degludec | A10AE06 |
|  | insulin degludec and insulin aspart | A10AD06 |
|  | insulin detemir | A10AE05 |
|  | insulin glargine | A10AE04 |
|  | insulin glulisine | A10AB06 |
|  | insulin lispro | A10AB04 |
|  | insulin lispro | A10AD04 |
| Dementia medication | rivastigmine | N06DA03 |
| (oral, topical) | donepezil | N06DA02 |
|  | galantamine | N06DA04 |
|  | memantine | N06DX01 |

ATC: Anatomical therapeutic chemical

Table S2. The composition of ambulatory care sensitive condition of patients aged 65 and older (N=85,582)

| ACSC category | ACSC | n | % | ICD-10 Codes |
| --- | --- | --- | --- | --- |
| Acute  (n = 28,315) | Cellulitis | 5,157 | 6.0 | L030-L033, L038, L039, L040-L043, L048, L049, L080, L088, L089, L980 |
|  | Dehydration and gastroenteritis | 8,051 | 9.4 | E86, K522, K528, K529 |
|  | Dental conditions | 81 | 0.1 | A690, A698, A699, K020-K025, K028, K029, K030-K039, K040-K049, K050-K056, K060-K062, K068, K069, K081-K083, K088, K089, K098 K099, K120-K123, K130-K137 |
|  | Ear, nose, and throat infections | 170 | 0.2 | H660-H664, H669, H670, H671, H678, J020, J028, J029, J30, J038, J039, J060, J068, J312 |
|  | Gangrene | 50 | 0.1 | R02 |
|  | Nutritional deficiency | 78 | 0.1 | E40, E41-E43, E550, E643 |
|  | Pelvic inflammatory infections | 53 | 0.1 | N700, N701, N709, N730-N736, N738, N739, E740-E744, E748 |
|  | Perforated or bleeding ulcer | 3,284 | 3.8 | K250-K252, K254-K256, K260-K262, K264-K266, K270-K272, K274-K276, K280-K282, K284-K286 |
|  | pyelonephritis | 11,391 | 13.3 | N10, N110, N111, N118, N119, N12, N136 |
| Chronic  (n = 48,116) | Angina | 4,854 | 5.7 | I200, I201, I208, I209, I240, I248, I249 |
|  | Asthma | 954 | 1.1 | J450, J451, J458-J460 |
|  | Chronic obstructive pulmonary disease | 2,765 | 3.2 | J200-J209, 410, J411, J418, J42, J430-J432, J438, J439, J440, J441, J448, J449, J47 |
|  | Congestive heart failure | 31,739 | 37.1 | I110, I500, I501, I509, J81 |
|  | Conversions and epilepsy | 3,754 | 4.4 | G400-G409, G410-G412, G418, G419, R560, R568, O150-O152, O159 |
|  | Diabetes complications | 2,569 | 3.0 | E100-E108, E110-E118, E120-E128, E130-E138, E140-E148 |
|  | Hypertension | 402 | 0.5 | I10, I119 |
|  | Iron-deficiency anaemia | 1,079 | 1.3 | D501, D508, D509 |
| Vaccine-preventable  (n = 9,151) | Influenza and pneumonia | 9,114 | 10.7 | J100, J101, J108, J110, J111, J118, J13, J14, J153, J154, J157, J159, J168, J181, J188 |
|  | Other vaccine-preventable diseases | 37 | 0.0 | A35, A360-A363, A368, A369, A370, A371, A378, A379, A800-A804, A809, B050-B054, B058, B050-B054, B058, B059, B060, B068, B069, B161, B169, B180, B181, B260-263, B268, B269, B180, B181, G000, M014 |

ACSC: Ambulatory care sensitive condition; ICD: International Statistical Classification of Diseases

Table S3. Characteristics of acute ambulatory care sensitive condition before and after inverse-probability treatment weighting (n=28,315)

|  | Before weighted | | | After weighted | | |
| --- | --- | --- | --- | --- | --- | --- |
|  | With transitional care | Without transitional care | Standard difference | With transitional care | Without transitional care | Standard difference |
|  | (n = 18,540) | (n = 9,775) |  | (n = 18,540) | (n = 9,775) |  |
| Female, n (%) | 9,903 (53.4) | 4,783 (48.9) | 0.12 | 9,577 (51.7) | 5,131 (47.5) | 0.00 |
| Age, median (IQR) | 83 (77-89) | 79 (73-86) | 0.35 | 82 (75-88) | 82 (75-88) | 0.02 |
| Location before hospitalisation, n (%) |  |  |  |  |  |  |
| Home | 15,317 (82.6) | 8,809 (90.1) | 0.17 | 15,680 (84.6) | 8,362 (85.6) | 0.02 |
| Transfer | 388 (2.1) | 143 (1.5) | 0.03 | 358 (1.9) | 169 (1.7) | 0.01 |
| Long-term care facility | 2,823 (15.2) | 819 (8.4) | 0.17 | 2,492 (13.4) | 1,237 (12.7) | 0.02 |
| Other | 12 (0.1) | 4 (0) | 0.01 | 10 (0.1) | 7 (0.1) | 0.00 |
| Body Mass Index, median (IQR) | 21.3 (18.5-24.1) | 22 (19.3-24.8) | 0.14 | 21.5 (18.7-24.3) | 21.5 (18.9-24.4) | 0.01 |
| Charlson Comorbidity Index, n (%) |  |  |  |  |  |  |
| 0 points | 6,993 (37.7) | 4,172 (42.7) | 0.15 | 7,335 (39.5) | 3,763 (38.5) | 0.01 |
| 1 point | 1,611 (8.7) | 942 (9.6) | 0.07 | 1,691 (9.1) | 869 (8.9) | 0.00 |
| 2 points | 6,167 (33.3) | 3,003 (30.7) | 0.05 | 6,034 (32.6) | 3,129 (32) | 0.00 |
| 3 points or more | 3,769 (20.3) | 1,658 (17) | 0.13 | 3,479 (18.8) | 2,013 (20.6) | 0.01 |
| Presence of dementia, n (%) | 4,422 (23.9) | 1,134 (11.6) | 0.23 | 3,758 (20.3) | 1,909 (19.5) | 0.03 |
| Use of ER or ICU, n (%) | 1,270 (6.9) | 473 (4.8) | 0.11 | 1,161 (6.3) | 573 (5.9) | 0.00 |
| Use of ventilator, n (%) | 12 (0.1) | 5 (0.1) | 0.03 | 11 (0.1) | 11 (0.1) | 0.00 |
| Use of dialysis, n (%) | 298 (1.6) | 164 (1.7) | 0.05 | 338 (1.8) | 134 (1.4) | 0.00 |
| Prescription of injectable antidiabetic medication, n (%) | 1,327 (7.2) | 605 (6.2) | 0.25 | 1,266 (6.8) | 701 (7.2) | 0.02 |
| Home care before hospitalisation, n (%) |  |  |  |  |  |  |
| No | 15,732 (84.9) | 9,112 (93.2) | 0.25 | 16,222 (87.5) | 8,575 (87.7) | 0.02 |
| Yes | 2,458 (13.3) | 626 (6.4) | 0.22 | 2,054 (11.1) | 1,108 (11.3) | 0.02 |
| Unknown | 350 (1.9) | 37 (0.4) | 0.11 | 264 (1.4) | 91.2 (0.9) | 0.00 |
| Status of Long-term care insurance, n (%) |  |  |  |  |  |  |
| None | 7,288 (39.3) | 6,307 (64.5) | 0.47 | 8,711 (47) | 4,722 (48.3) | 0.02 |
| In application or certified | 10,864 (58.6) | 3,256 (33.3) | 0.48 | 9,416 (50.8) | 4,876 (49.9) | 0.02 |
| Unknown | 388 (2.1) | 212 (2.2) | 0.03 | 413 (2.2) | 176 (1.8) | 0.00 |
| Type of ownership, n (%) |  |  |  |  |  |  |
| National | 2,128 (11.5) | 1,323 (13.5) | 0.04 | 2,221 (12) | 1,212 (12.4) | 0.00 |
| Public | 9,306 (50.2) | 3,990 (40.8) | 0.23 | 8,621 (46.5) | 4,641 (47.5) | 0.01 |
| Social | 503 (2.7) | 209 (2.1) | 0.04 | 464 (2.5) | 230 (2.4) | 0.00 |
| Private | 5,904 (31.8) | 3,747 (38.3) | 0.16 | 6,344 (34.2) | 3,326 (34) | 0.00 |
| Others | 699 (3.8) | 506 (5.2) | 0.13 | 890 (4.8) | 365 (3.7) | 0.00 |
| Accreditation as a regional medical care support hospital, n (%) | 12,116 (65.4) | 5,109 (52.3) | 0.27 | 11,323 (61.1) | 5,933 (60.7) | 0.01 |
| Accreditation as a home medical care support hospital, n (%) | 5,518 (29.8) | 2,542 (26) | 0.09 | 5,238 (28.3) | 2,864 (29.3) | 0.01 |
| Number of hospital beds, median (IQR) | 404 (300-535) | 394 (283-556) | 0.02 | 400 (299-535) | 400 (292-554) | 0.01 |
| Number of doctors per 100 beds, median (IQR) | 26 (19.8-33.8) | 26 (19.5-35.5) | 0.06 | 26 (19.7-34.2) | 26 (19.7-33.9) | 0.01 |
| Number of nursing staff per 100 beds, median (IQR) | 101 (88.5-113.7) | 102 (88.3-163.3) | 0.02 | 101 (88.5-113.8) | 102 (88.3-113.7) | 0.01 |
| Number of nursing staff and MSWs in discharge planning department per 100 beds, median (IQR) | 2.6 (1.9-3.6) | 2.4 (1.8-3.4) | 0.07 | 2.6 (2.0-3.5) | 2.5 (1.7-3.6) | 0.01 |
| Population (100,000 people), median (IQR) | 5.7 (2.0-13.9) | 5.7 (2.0-14.5) | 0.01 | 5.8 (2.0-13.9) | 5.7 (1.9-13.9) | 0.00 |
| Percentage of individuals aged 65 years and older, median (IQR) | 31 (26.8-36.7) | 31.5 (26.8-36.3) | 0.01 | 30.8 (26.8-36.7) | 31.5 (26.9-36.3) | 0.01 |
| Number of home health care support clinics per 10,000 people aged 65 years and older, median (IQR) | 3.3 (1.1-13.5) | 3.1 (1.1-8.8) | 0.10 | 3.2 (1.1-12.0) | 3.2 (1.1-11.4) | 0.01 |
| Number of in-home service agencies per 10,000 people aged 65 years and older, median (IQR) | 216.9 (93.3-656.3) | 210.5 (87.3-654.5) | 0.03 | 216.9 (94.3-654.5) | 216.8 (92.4-654.5) | 0.01 |

IQR; Interquartile range; ER: Emergency room; ICU: Intensive care unit; MSW: Medical social worker

Table S4. Characteristics of chronic ambulatory care sensitive condition before and after inverse-probability treatment weighting (n=48,116)

|  | Before weighted | | | After weighted | | |
| --- | --- | --- | --- | --- | --- | --- |
|  | With transitional care | Without transitional care | Standard difference | With transitional care | Without transitional care | Standard difference |
|  | (n = 32,598) | (n = 15,518) |  | (n = 32,598) | (n = 15,518) |  |
| Female, n (%) | 15,817 (48.5) | 6,302 (40.6) | 0.12 | 15,083 (46.3) | 7,136 (46.0) | 0.01 |
| Age, median (IQR) | 84 (77-89) | 80 (74-86) | 0.35 | 84 (77-89) | 80 (74-86) | 0.02 |
| Location before hospitalisation, n (%) |  |  |  |  |  |  |
| Home | 28,374 (87.0) | 14,198 (91.5) | 0.17 | 28,889 (88.6) | 13,517 (87.1) | 0.02 |
| Transfer | 815 (2.5) | 350 (2.3) | 0.03 | 771 (2.4) | 426 (2.7) | 0.01 |
| Long-term care facility | 3,390 (10.4) | 961 (6.2) | 0.17 | 2,920 (9.0) | 1,564 (10.1) | 0.02 |
| Other | 19 (0.1) | 9 (0.1) | 0.01 | 18 (0.1) | 10 (0.1) | 0.00 |
| Body Mass Index, median (IQR) | 22 (19.0-24.3) | 22 (19.8-24.9) | 0.14 | 22 (19.2-24.5) | 22 (19.4-24.5) | 0.05 |
| Charlson Comorbidity Index, n (%) |  |  |  |  |  |  |
| 0 points | 2,844 (8.7) | 2,495 (16.1) | 0.15 | 3,140 (9.6) | 2,048 (13.2) | 0.01 |
| 1 point | 2,061 (6.3) | 1,334 (8.6) | 0.07 | 2,273 (7.0) | 1,139 (7.3) | 0.00 |
| 2 points | 13,609 (41.8) | 6,108 (39.4) | 0.05 | 13,730 (42.1) | 6,086 (39.2) | 0.00 |
| 3 points or more | 14,084 (43.2) | 5,581 (36.0) | 0.13 | 13,455 (41.3) | 6,244 (40.2) | 0.01 |
| Presence of dementia, n (%) | 5,245 (16.1) | 1,346 (8.7) | 0.26 | 4,428 (13.6) | 2,374 (15.3) | 0.03 |
| Use of ER or ICU, n (%) | 8,447 (25.9) | 3,324 (21.4) | 0.11 | 7,932 (24.3) | 3,846 (24.8) | 0.00 |
| Use of ventilator, n (%) | 508 (1.6) | 192 (1.2) | 0.03 | 476 (1.5) | 241 (1.6) | 0.00 |
| Use of dialysis, n (%) | 1,192 (3.7) | 790 (5.1) | 0.05 | 1,318 (4.0) | 652 (4.2) | 0.00 |
| Prescription of injectable antidiabetic medication, n (%) | 3,799 (11.7) | 1,556 (10.0) | 0.05 | 3,647 (11.2) | 1,740 (11.2) | 0.01 |
| Home care before hospitalisation, n (%) |  |  |  |  |  |  |
| No | 28,384 (87.1) | 14,573 (93.9) | 0.25 | 29,113 (89.3) | 13,712 (88.4) | 0.02 |
| Yes | 3,767 (11.6) | 874 (5.6) | 0.22 | 3,143 (9.6) | 1,606 (10.4) | 0.02 |
| Unknown | 447 (1.4) | 71 (0.5) | 0.11 | 342 (1.1) | 200 (1.3) | 0.00 |
| Status of Long-term care insurance, n (%) |  |  |  |  |  |  |
| None | 14,523 (44.6) | 10,216 (65.8) | 0.47 | 16,880 (51.8) | 7,715 (49.7) | 0.02 |
| In application or certified | 17,419 (53.4) | 4,873 (31.4) | 0.48 | 15,021 (46.1) | 7,422 (47.8) | 0.02 |
| Unknown | 656 (2.0) | 429 (2.8) | 0.03 | 696 (2.1) | 382 (2.5) | 0.00 |
| Type of ownership, n (%) |  |  |  |  |  |  |
| National | 4,174 (12.8) | 2,048 (13.2) | 0.04 | 4,323 (13.3) | 2,010 (13.0) | 0.00 |
| Public | 15,559 (47.7) | 5,492 (35.4) | 0.23 | 14,275 (43.8) | 6,689 (43.1) | 0.01 |
| Social | 945 (2.9) | 374 (2.4) | 0.04 | 872 (2.7) | 442 (2.9) | 0.00 |
| Private | 10,875 (33.4) | 6,490 (41.8) | 0.16 | 11,761 (36.1) | 5,590 (36.0) | 0.00 |
| Others | 1,045 (3.2) | 1,114 (7.2) | 0.23 | 1,368 (4.2) | 788 (5.1) | 0.00 |
| Accreditation as a regional medical care support hospital, n (%) | 22,779 (69.9) | 8,875 (57.2) | 0.27 | 21,333 (65.5) | 10,265 (66.2) | 0.02 |
| Accreditation as a home medical care support hospital, n (%) | 8,019 (24.6) | 3,098 (20.0) | 0.09 | 7,615 (23.4) | 3,559 (22.9) | 0.01 |
| Number of hospital beds, median (IQR) | 434 (315-578) | 414 (297-574) | 0.02 | 426 (313-578) | 422 (302-572) | 0.01 |
| Number of doctors per 100 beds, median (IQR) | 29 (20.9-36.5) | 28 (19.8-36.9) | 0.06 | 29 (20.5-36.8) | 28 (20.2-36.7) | 0.01 |
| Number of nursing staff per 100 beds, median (IQR) | 104 (91.7-114.2) | 104 (90-116.1) | 0.02 | 103 (90.9-114.2) | 104 (90.5-115.7) | 0.01 |
| Number of nursing staff and MSWs in discharge planning department per 100 beds, median (IQR) | 2.5 (1.9-3.4) | 2.4 (1.8-3.4) | 0.07 | 2.5 (1.9-3.4) | 2.5 (1.8-3.5) | 0.01 |
| Population (100,000 people), median (IQR) | 7.0 (2.2-15) | 5.8 (1.9-15) | 0.01 | 7.1 (2.2-15.0) | 5.9 (2.0-15.0) | 0.00 |
| Percentage of individuals aged 65 years and older, median (IQR) | 30.5 (26.4-36) | 31.0 (26.8-36.2) | 0.01 | 30.5 (26.4-36.0) | 30.1 (26.8-36.2) | 0.01 |
| Number of home health care support clinics per 10,000 people aged 65 years and older, median (IQR) | 3.2 (1.1-12) | 3.0 (1.1-8.8) | 0.10 | 3.0 (1.1-11.7) | 3.1 (1.1-11.4) | 0.01 |
| Number of in-home service agencies per 10,000 people aged 65 years and older, median (IQR) | 215.5 (94.2-654.5) | 216.8 (94.2-573.9) | 0.03 | 210.5 (94.3-555.8) | 216.8 (94.2-633.4) | 0.10 |

IQR; Interquartile range; ER: Emergency room; ICU: Intensive care unit; MSW: Medical social worker

Table S5. Characteristics of vaccine-preventable ambulatory care sensitive condition before and after inverse-probability treatment weighting (n=9,151)

|  | Before weighted | | | After weighted | | |
| --- | --- | --- | --- | --- | --- | --- |
|  | With transitional care | Without transitional care | Standard difference | With transitional care | Without transitional care | Standard difference |
|  | (n = 3,162) | (n = 5,989) |  | (n = 5,989) | (n = 3,162) |  |
| Female, n (%) | 2,102 (35.1) | 1,009 (31.9) | 0.12 | 1,994 (33.3) | 1,080 (34.2) | 0.00 |
| Age, median (IQR) | 83 (77-88) | 79 (73-85) | 0.35 | 82 (75-87) | 81 (75-87) | 0.02 |
| Location before hospitalisation, n (%) |  |  |  |  |  |  |
| Home | 5,118 (85.5) | 2,878 (91.0) | 0.17 | 5,227 (87.3) | 2,740 (86.6) | 0.02 |
| Transfer | 122 (2.0) | 38 (1.2) | 0.03 | 111 (1.9) | 43 (1.4) | 0.01 |
| Long-term care facility | 743 (12.4) | 245 (7.8) | 0.17 | 645 (10.8) | 378 (12.0) | 0.02 |
| Other | 6 (0.1) | 1 (0.0) | 0.01 | 6 (0.1) | 1 (0.0) | 0.00 |
| Body Mass Index, median (IQR) | 20 (17.7-23.1) | 21 (18.3-23.5) | 0.14 | 21 (17.9-23.3) | 21 (17.9-23.2) | 0.01 |
| Charlson Comorbidity Index, n (%) |  |  |  |  |  |  |
| 0 points | 1,524 (25.5) | 819 (25.9) | 0.15 | 1,591 (26.6) | 760 (24.0) | 0.01 |
| 1 point | 931 (15.6) | 596 (18.9) | 0.07 | 994 (16.6) | 513 (16.2) | 0.00 |
| 2 points | 1,702 (28.4) | 861 (27.2) | 0.05 | 1,659 (27.7) | 876 (27.7) | 0.00 |
| 3 points or more | 1,832 (30.6) | 886 (28.0) | 0.13 | 1,744 (29.1) | 1,013 (32.0) | 0.00 |
| Presence of dementia, n (%) | 1,185 (19.8) | 349 (11.0) | 0.26 | 1,003 (16.8) | 580 (18.3) | 0.03 |
| Use of ER or ICU, n (%) | 551 (9.2) | 214 (6.8) | 0.10 | 493 (8.2) | 271 (8.6) | 0.00 |
| Use of ventilator, n (%) | 36 (0.6) | 12 (0.4) | 0.03 | 32 (0.5) | 12 (0.4) | 0.00 |
| Use of dialysis, n (%) | 153 (2.6) | 127 (4.0) | 0.05 | 169 (2.8) | 101 (3.2) | 0.02 |
| Prescription of injectable antidiabetic medication, n (%) | 464 (7.8) | 234 (7.4) | 0.05 | 440 (7.4) | 275 (8.7) | 0.01 |
| Home care before hospitalisation, n (%) |  |  |  |  |  |  |
| No | 5,127 (85.6) | 2,944 (93.1) | 0.25 | 5,272 (88.0) | 2,763 (87.4) | 0.02 |
| Yes | 784 (13.1) | 201 (6.4) | 0.22 | 658 (11.0) | 371 (11.7) | 0.02 |
| Unknown | 78 (1.3) | 17 (0.5) | 0.11 | 58 (1.0) | 28 (0.9) | 0.00 |
| Status of Long-term care insurance, n (%) |  |  |  |  |  |  |
| None | 2,600 (43.4) | 2,084 (65.9) | 0.47 | 3,053 (51.0) | 1,585 (50.1) | 0.02 |
| In application or certified | 3,256 (54.4) | 1,004 (31.8) | 0.22 | 2,791 (46.6) | 1,512 (47.9) | 0.02 |
| Unknown | 133 (2.2) | 74 (2.3) | 0.03 | 144 (2.4) | 63 (2.0) | 0.00 |
| Type of ownership, n (%) |  |  |  |  |  |  |
| National | 823 (13.7) | 547 (17.3) | 0.04 | 867 (14.5) | 497 (15.7) | 0.00 |
| Public | 3,042 (50.8) | 1,220 (38.6) | 0.23 | 2,833 (47.3) | 1,430 (45.2) | 0.01 |
| Social | 190 (3.2) | 65 (2.1) | 0.04 | 176 (2.9) | 72 (2.3) | 0.00 |
| Private | 1,704 (28.5) | 1,184 (37.4) | 0.16 | 1,822 (30.4) | 1,049 (33.2) | 0.00 |
| Others | 230 (3.8) | 146 (4.6) | 0.12 | 291 (4.9) | 115 (3.6) | 0.00 |
| Accreditation as a regional medical care support hospital, n (%) | 4,115 (68.7) | 1,732 (54.8) | 0.27 | 3,870 (64.6) | 1,942 (61.5) | 0.00 |
| Accreditation as a home medical care support hospital, n (%) | 1,646 (27.5) | 777 (24.6) | 0.09 | 1,585 (26.5) | 876 (27.7) | 0.01 |
| Number of hospital beds, median (IQR) | 416 (301-569) | 405 (282-576) | 0.02 | 411 (300-570) | 418 (292-574) | 0.01 |
| Number of doctors per 100 beds, median (IQR) | 27 (20.5-35.3) | 27 (19.8-35.4) | 0.06 | 27 (20.3-35.5) | 27 (20.2-35.3) | 0.01 |
| Number of nursing staff per 100 beds, median (IQR) | 102 (90.8-114.4) | 104 (89.1-116.4) | 0.02 | 102 (90.5-114.5) | 103 (89.5-115.1) | 0.01 |
| Number of nursing staff and MSWs in discharge planning department per 100 beds, median (IQR) | 2.6 (2-3.4) | 2.4 (1.8-3.4) | 0.08 | 2.5 (1.9-3.4) | 2.5 (1.8-3.5) | 0.01 |
| Population (100,000 people), median (IQR) | 5.9 (2-13.9) | 5.9 (2.04-13.5) | 0.01 | 5.9 (2.0-13.5) | 5.9 (2.0-13.9) | 0.00 |
| Percentage of individuals aged 65 years and older, median (IQR) | 30.7 (26.4-36.2) | 31.0 (26.8-36) | 0.01 | 30.7 (26.4-36.2) | 31.0 (26.8-36.0) | 0.01 |
| Number of home health care support clinics per 10,000 people aged 65 years and older, median (IQR) | 3.0 (1.1-11.3) | 3.0 (1.2-8.6) | 0.10 | 3.1 (1.1-10.8) | 3.1 (1.1-8.8) | 0.01 |
| Number of in-home service agencies per 10,000 people aged 65 years and older, median (IQR) | 216.8 (93.3-654.5) | 212.1 (92.5-654.5) | 0.03 | 215.5 (94.2-654.5) | 215.5 (92.5-654.5) | 0.01 |

IQR; Interquartile range; ER: Emergency room; ICU: Intensive care unit; MSW: Medical social worker

Table S6. Actual and predicted readmission rates by ambulatory care sensitive condition category (N=85,582)

|  | Acute ACSC (n = 28,315) | | | | Chronic ACSC (n = 48,116) | | | | Vaccine-preventable (n = 9,151) | | | |
| --- | --- | --- | --- | --- | --- | --- | --- | --- | --- | --- | --- | --- |
|  | With TC (n = 18,540) | | Without TC (n = 9,775) | | With TC (n = 32,598) | | Without TC (n = 15,518) | | With TC (n = 5,989) | | Without TC (n = 3,162) | |
|  | Actual | Predict | Actual | Predict | Actual | Predict | Actual | Predict | Actual | Predict | Actual | Predict |
| 7 days, % | 2.5 | 2.4 | 3.1 | 3.2 | 2.8 | 2.8 | 3.5 | 3.8 | 3.3 | 3.2 | 4.3 | 5.0 |
| 14 days, % | 4.8 | 4.7 | 5.5 | 5.7 | 6.3 | 6.3 | 7.2 | 7.6 | 5.5 | 5.5 | 6.7 | 7.4 |
| 21 days, % | 6.6 | 6.6 | 7.9 | 8.0 | 9.4 | 9.5 | 10.3 | 10.6 | 7.8 | 7.8 | 8.6 | 9.1 |
| 30 days, % | 8.7 | 8.6 | 10.2 | 10.5 | 12.6 | 12.7 | 13.4 | 13.7 | 9.9 | 9.9 | 10.6 | 11.2 |
| 60 days, % | 13.3 | 13.1 | 15.1 | 15.3 | 19.8 | 19.9 | 20.7 | 20.8 | 14.5 | 14.4 | 15.7 | 16.1 |

ACSC: Ambulatory care sensitive condition; TC: Transitional care

The predicted readmission rates were calculated from the models estimated with inverse probability treatment weighting analysis.

Table S7. E-value for readmission rates due to overall ambulatory care sensitive condition (N=85,582)

|  | OR (95%CI) | E-value for OR | E-value for CI lower limit |
| --- | --- | --- | --- |
| 7 days | 0.72 (0.65-0.78) | 2.12 | 1.88 |
| 14 days | 0.81 (0.76-0.86) | 1.77 | 1.60 |
| 21 days | 0.86 (0.81-0.91) | 1.6 | 1.43 |
| 30 days | 0.88 (0.84-0.93) | 1.53 | 1.36 |
| 60 days | 0.91 (0.87-0.95) | 1.27 | 1.19 |

OR; Odds ratio; CI: Confidence interval
